# The positivity bias of Chinese temporal collective self: Evidence from the first-person perspective and the third-person perspective

**DOI:** 10.3389/fpsyg.2023.1060068

**Published:** 2023-03-09

**Authors:** Caizhen Yue, Yihong Long, Chaomei Ni, Huating Wu, Dexuan Zhao

**Affiliations:** ^1^College of National Culture and Cognitive Science, Guizhou Minzu University, Guiyang, China; ^2^Faculty of Psychology, Beijing Normal University, Beijing, China

**Keywords:** temporal collective self, positive bias, first-person perspective, third-person perspective, mental time travel

## Abstract

**Objective:**

As a unique part of human thinking, people can project themselves into the past or the future for mental time travel. This study attempts to expand the temporal self into the domain of the collective self.

**Methods:**

We used an adapted temporal collective self-reference paradigm to probe into the positivity bias of temporal collective self in this study. In Experiment 1, the first-person perspective was adopted for the participants to conduct the temporal collective self-reference processing, and the third-person perspective was adopted for the temporal collective self-reference processing in Experiment 2.

**Results:**

The findings indicated that no matter from the first-person perspective or the third-person perspective, people show positivity bias in the trait adjectives judgment, response times and recognition rates during the temporal collective self-processing.

**Discussion:**

This study explores mental time travel on the level of collective self, and contributes to deepening the understanding of temporal collective self.

## Introduction

The self-system consists of three basic components: collective self, relational self and individual self. Different from the individual self which reflects the uniqueness of the individual, and the relational self which reflects the relationship between the individual and the intimate others, the collective self mainly emphasizes the relationship between the self and the subordinate group. The collective self means that people can define and understand selves in terms of group membership, which specifically reflects the membership in a valuable social group, as well as the similarity and identity with the group (Sedikides et al., 2013). For example, people usually regard themselves as members of the nation to represent the collective self (Zheng et al., 2018). This is similar to the research results of the individual self, then it is found that compared with other information, relevant information of the collective self has higher emotional significance, reward value and more familiarity (Chen et al., 2011), and it is closely related to people’s mental and physical health (Sheldon and Filak, 2008; Haslam et al., 2009). As a unique part of human thinking, people can project themselves into the past or the future for mental time travel (MTT) (Tulving, 2002). Generally, people can re-experience their past by subjectively self-positioning to the time and place they have experienced before, or by self-positioning to a future time point to experience certain events (Liu et al., 2018). Existing studies on the temporal self focus more on the individual self. As the collective self that is equally important and meaningful to the individual, can people conduct mental time travel at the collective level? It is found that although previous studies have shown that personal and collective cognition has a lot of similarities (Caruso, 2010; Peetz et al., 2010; Caouette et al., 2012; Wildschut et al., 2014), but people’s time orientation at the collective level can be distinguished from that at the individual level. Collective time orientation can be a meaningful structure independent of individual time orientation. Moreover, people show different collective time orientations for different collective types (Peetz and Wohl, 2019).

Through mental time travel, individuals can perceive past self, present self and future self as a continuous whole, promoting the formation of stable self-identity of individuals (Northoff, 2017). Similar to the individual self, people will also show collective continuity when conducting the temporal collective self-processing, in other words, people usually think that the collectives at different time stages are connected (Sani et al., 2007; Warner et al., 2016). This perceived collective continuity brings people higher collective self-esteem, higher happiness, and higher collective identity (Smeekes and Verkuyten, 2014; Goto et al., 2015; Warner et al., 2016; Siromahov et al., 2020; Maoulida et al., 2021). On the one hand, people regard the past, present and future collectives as a unified and continuous whole. On the other hand, what are the differences and connections among the three parts (past collective self, present collective self, and future collective self) of the temporal collective self? Researchers have mainly explored the correlation between the past collective self (focusing on the theme of collective memory) and the future collective self (focusing on the theme of collective future imagination) (Merck et al., 2016; Szpunar and Szpunar, 2016; Hirst et al., 2018).

People’s cognitive processes of collective memory and collective future imagination are not completely consistent (Michaelian and Sutton, 2019). Collective memory provides the basis for collective future imagination, meanwhile, collective future imagination will in turn shape the way that the collective past is remembered (Szpunar and Szpunar, 2016; De Saint-Laurent, 2018). The study also found that compared with collective memory, people’s reactions to the collective future are less specific, and people think they have more control over the collective future (Topcu and Hirst, 2020). The study suggested that the past collective self and the future collective self, respectively, show certain uniqueness.

Research on the self has found that people often show a more positivity bias when they conduct self-process (Watson et al., 2007; Zhou et al., 2013), which means people are usually inclined to view themselves with a positive attitude. Researchers have verified the stability of the positivity bias of self from different perspectives. People generally rate positive traits as self-relevant and negative traits as self-irrelevant, and perceive themselves as having much more positive traits (and fewer negative traits) and abilities (Fields et al., 2019); Positive traits or outcomes are attributed to internal, stable and holistic personality traits (Herbert et al., 2008). Evidence of self-positivity bias has also been found in self-relevant information processing in cognitive psychology. Self-positivity bias allows individuals to select and filter input information, and individuals respond more quickly to trait adjectives within the scope of self-positivity bias than to those outside the scope. For example, Chen et al. (2014) adopted the self-reference paradigm to ask the participants to judge personality trait adjectives, and found that individuals respond more quickly to self-positive adjectives than to self-negative adjectives. Self-positivity bias not only shows in the individual self, but also in the collective self. For example, people usually show a preference for in-groups (classical social identity theory) (Tajfel et al., 1979) and tend to link in-groups with positive adjectives but out-groups with negative adjectives (Lyu et al., 2019). Therefore, we predict that the self-positivity bias is also reflected in the temporal collective self, that is, the past collective self, present collective self, and future collective self all show positivity bias (Hypothesis 1).

While people judge themselves positively at different points in time, the degree of self-positivity may vary from different points in time. According to Temporal Self-appraisal Theory (Wilson and Ross, 2001) holds that people usually tend to devalue the past self but value the future self from a more positive perspective, which has been supported by numerous studies in the field of the individual self (Hershfield, 2011; Szpunar et al., 2012; Sokol and Serper, 2017; Yang et al., 2017; Yue et al., 2021). For instance, positive future events are remembered in more detail than negative future events (Gallo et al., 2011), and future events are rated as more emotionally positive than past events (Berntsen and Jacobsen, 2008; Berntsen and Bohn, 2010). Even individuals with depressed emotions are optimistic about their future selves (depression and hopelessness). These studies show that people are most positive about their future selves. Based on this, we predict that Temporal Self-appraisal Theory should also be extended to the field of the collective self, that is, compared with the past collective self, people should show a more positivity bias toward the future collective self (Hypothesis 2).

In the process of self-knowledge, people can view themselves not only from their own point of view (i.e., first-person perspective), but also from the perspective of external others (i.e., third-person perspective). According to the Construal Level Theory (CLT) (Trope and Liberman, 2010), people can think about the past, the future, or view themselves from the perspective of others, all of which have psychological distance, but which make up different types of traversing psychological distance. In other words, psychological distance is egocentric, whose reference point is the self under the present circumstances. Individuals can move away from this point in time, space, and social distance, constituting different distance dimensions. The self beyond the present circumstances needs psychological construal, and short-distance events are relevant to low-level concrete construal, including complex, non-structured and contextualized expressions. However, long-distance events are relevant to high-level abstract construal, including abstract, schematic and non-textual expressions (Trope and Liberman, 2003; Henderson et al., 2011). It is found that, like time, the third-person perspective usually has the distancing function (D'Argembeau and Van der Linden, 2004; Sutin and Robins, 2008). Therefore, when people process the past self and the future self, or adopt the third-person perspective, they usually explain events at the level of generalization or abstraction (Liberman et al., 2002; Libby et al., 2005; Wakslak et al., 2008). For example, in comparison with the first-person perspective, making observations on a person’s behaviors from the third-person perspective may enable people to evaluate their own behaviors more objectively (Zhou et al., 2013), to have lower emotional experience (Berntsen and Rubin, 2006), and to reduce egocentric bias (Zhou et al., 2013). In the study of the temporal self at the individual level, it is found that people usually view long-distance events from the third-person perspective (Pronin and Ross, 2006; Zhou et al., 2013). Since people have a strong positivity bias in self-knowledge, we predict that this positivity bias will not only be manifested under the first-person perspective, but also in the collective self under the third-person perspective (Hypothesis 3), and that people will also show a more positivity bias toward the future collective self under the third-person perspective (Hypothesis 4).

In order to discuss the positivity bias of the temporal collective self, we adapted the classical self-reference paradigm, which usually includes three stages: encoding, interference, and recognition. In the encoding phase, the participants were asked to rate their own or others’ personality traits, followed by interference with unrelated tasks (such as Raven’s Progressive Matrices, RPM), and finally performed a recognition task. Researchers usually analyze trait adjectives rating scores, as well as response times and recognition rates of trait adjectives judgment to reveal the characteristics of self-reference processing (Conway and Dewhurst, 1995; Zhu and Zhang, 2002; Ketay et al., 2019;Yue et al., 2020). In addition, by referring to previous studies, we chose to use Chinese people to represent the collective self (Han et al., 2016; Zheng et al., 2018). Based on this, this study adopted the adapted temporal collective self-reference paradigm and explored the positivity bias of Chinese people’s temporal collective self from the first-person perspective (Experiment 1) and the third-person perspective (Experiment 2).

## Experiment 1

Experiment 1 aims to adopt the first-person perspective to explore the positivity bias of the temporal collective self. The participants were first asked to make trait adjectives judgments about the past collective self (Chinese five years ago), the present collective self (Chinese present), and the future collective self (Chinese five years later) from the first-person perspective, and then performed a recognition task. We predict that, no matter in trait adjectives rating scores, response times, or recognition rates, the temporal collective self in the past, present and future shows a positivity bias, and the future collective self may show a greater positivity bias.

### Methods

#### Participants

In this study, we used *f* = 0.27 as G-Power 3.1.9 (*α* = 0.05; Faul et al., 2007) to test the moderate impact of the primary outcome, and the finding indicated that the required sample size for Experiment 1 and Experiment 2 was 24 persons. 36 undergraduate students from Guizhou Minzu University were randomly selected to participate in Experiment 1 by convenient sampling (including 11 males and 25 females, and their average ages are 19.69 years old; SD = 1.01). All participants signed the written informed consent, which was approved by the Ethics Committee of Guizhou Minzu University.

#### Materials

The experimental materials and procedures of the two experiments in this study were the same as those of previous researches. The experimental materials were 240 personality trait adjectives (each consisting of 2–4 Chinese characters) selected from the rated Chinese personality trait adjectives database of Wang (2005). The trait adjectives were divided into 6 groups (40 adjectives in each group, 20 positive adjectives, such as generous and optimistic; and 20 negative adjectives, such as rude and timid). Pleasure, meaningfulness, familiarity, and the number of Chinese characters in each group were balanced (Yue et al., 2020). Three groups of trait adjectives were randomly selected as the learning stage for judgment (each group was randomly assigned to three encoding conditions: past collective self, present collective self and future collective self), and the other three groups of trait adjectives were selected as the new items in the recognition stage.

Referring to previous studies on temporal self, we used the IOS scale to measure the closeness of the temporal collective self (Aron et al., 1991). The IOS scale is made up of seven pairs of overlapping circles, each pair overlaps slightly more than the previous pair. The participants were asked to choose the pair of circles that best represented the relationship between the past (or future) collective self and the present collective self. Meanwhile, we also asked participants to rate the frequency of they recalled their past collective self or imagined the future collective self by using the seven-point rating scale (1 for never and 7 for very frequent).

### Procedures

After coming to the lab, the participants filled in the IOS scale and the rating frequency questionnaire first. The participants then performed a temporal collective self-reference task.

According to the self-reference paradigm, the experiment was divided into three stages: encoding, interference, and recognition.

First, the participants were asked to spend a minute thinking about (5 years ago/now/5 years later) Chinese people and then did an encoding task. After understanding the instruction and practicing the task of trait adjectives judgment, the participants randomly coded trait adjectives under three experimental conditions (past collective self, present collective self, and future collective self). Trait adjectives were presented in the order of the experimental tasks, and the presentation time of each trait adjective was 4,000 ms (the participants immediately entered the next adjective after pressing the button). The participants pressed the button to conduct a five-level evaluation on the trait adjective.

After the encoding phase, the participants were interfered with by taking Raven’s Progressive Matrices for 6 min. After the interference phase, the participants were given instructions to perform an unexpected recognition test. The computer randomly presented 120 trait adjectives which have been presented and 120 trait adjectives which have not appeared in the encoding stage after mixing them. The participants were asked to judge whether the trait adjective had appeared in the encoding stage (press F key) or whether the new adjective had not appeared (press J key). The 240 trait adjectives were processed in sequence, and there was no time limit for the recognition phase. Experimental tasks were presented through E-prime software. In this study, we analyzed the positivity bias of the temporal collective self through three indicators: rating scores in the encoding stage, response times, and recognition rates in the recognition phase.

### Results

#### IOS rating and frequency rating

The results of the paired sample t-test found no significant difference between the past-present collective self (*M* = 4.53, SD = 1.06) and the present-future collective self (*M* = 4.50, SD = 1.28), *t*(35) = 0.15, *p* > 0.05.

The results of the paired sample t-test showed no significant difference in the frequency between imagining the future collective self (*M* = 4.14, SD = 1.20) and recalling the past collective self (*M* = 4.28, SD = 1.43), *t*(35) = −0.63, *p* > 0.05.

#### Trait adjectives rating scores

The 2 (valence: positive/negative) × 3 (encoding condition: past/present/future collective self) repeated measures ANOVA was performed for trait adjectives rating scores (Seen in Table 1). The findings showed the valence of trait adjectives had a significant main effect [*F*(1,35) = 267.99, *p* < 0.001, $\eta_{p}^{2}$ =0.88]. The encoding condition had a significant main effect [*F*(2,70) = 3.827, *p* < 0.05, $\eta_{p}^{2}$ =0.10]. The valence and the encoding condition also had a significant interaction effect [*F*(2,70) = 5.59, *p* < 0.01, $\eta_{p}^{2}$ =0.14]. The simple effect analysis found that, under three kinds of encoding conditions, people gave significantly higher positive adjectives scores than negative adjectives scores, the past collective self [F(1,35) = 164.97, *p* < 0.001, $\eta_{p}^{2}$ =0.83], the present collective self [*F*(1,35) = 170.94, *p* < 0.001, $\eta_{p}^{2}$ =0.83], and the future collective self [*F*(1,35) = 452.36, *p* < 0.001, $\eta_{p}^{2}$ =0.93]. In rating positive trait adjectives, the scores of the future collective self were significantly higher than those of the past collective self and the present collective self (*ps* < 0.05), and there was no significant difference between the past collective self and the present collective self (*p* > 0.05).

**Table 1:** Descriptive statistical results of trait adjectives rating scores, response times, and recognition rates (*M* ± SD).

			Past collective self	Present collective self	Future collective self
Trait adjectives rating scores	Experiment 1	Positive	3.45(0.47)	3.53(0.49)	3.65(0.36)
		Negative	1.72(0.41)	1.78(0.40)	1.66(0.26)
	Experiment 2	Positive	3.42(0.44)	3.50(0.31)	3.57(0.36)
		Negative	1.65(0.44)	1.62(0.34)	1.59(0.30)
Response times (ms)	Experiment 1	Positive	1518.61(506.79)	1396.77(470.89)	1289.01(524.31)
		Negative	1683.75(471.99)	1602.74(530.01)	1510.24(481.10)
	Experiment 2	Positive	1789.32(626.17)	1637.67(434.89)	1575.62(553.44)
		Negative	1890.67(631.92)	1746.50(518.69)	1754.15(656.13)
Recognition rates	Experiment 1	Positive	0.39(0.08)	0.40(0.08)	0.40(0.08)
		Negative	0.34(0.11)	0.34(0.11)	0.34(0.09)
	Experiment 2	Positive	0.36(0.09)	0.36(0.08)	0.37(0.08)
		Negative	0.35(0.09)	0.31(0.12)	0.34(0.09)

#### Response times

The 2 (valence: positive/negative) × 3 (encoding condition: past/present/future collective self) repeated measures ANOVA was performed for response times during the encoding phase. The results presented the main effect of the valence was significant [*F*(1,35) = 35.16, *p* < 0.001, $\eta_{p}^{2}$ =0.50], and the negative response times (*M* = 1598.91, SD = 72.69) were significantly longer than positive (*M* = 1401.46, SD = 76.87) (*p* < 0.05). The encoding condition had the significant main effect [*F*(2,70) = 6.69, *p* < 0.01, $\eta_{p}^{2}$ =0.16]. The valence and the encoding condition had no significant interaction effect [*F*(2,70) = 0.49, *p* = 0.61, $\eta_{p}^{2}$ = 0.01]. Under the three kinds of encoding conditions, the negative response times were significantly longer than the positive response times, the past collective self [*F*(1,35) = 18.12, *p* < 0.001, $\eta_{p}^{2}$ =0.34], the present collective self [*F*(1,35) = 13.91, *p* < 0.001, $\eta_{p}^{2}$ = 0.28], and the future collective self [*F*(1,35) = 22.48, *p* < 0.001, $\eta_{p}^{2}$ = 0.39].

#### Recognition rates

The 2 (valence: positive/negative) × 3 (encoding condition: past/present/future collective self) repeated measures ANOVA was performed on the recognition rates during the recognition phase. The findings showed the valence had a significant main effect [*F*(1,35) = 20.24, *p* < 0.001, $\eta_{p}^{2}$ = 0.37], and the recognition rates of positive trait adjectives (*M* = 0.40, SD = 0.01) were significantly higher than those of negative (*M* = 0.35, SD = 0.02) (*p* < 0.001). The encoding condition had no significant main effect [*F*(2,70) = 0.01, *p* = 0.99, $\eta_{p}^{2}$ =0.00]. The valence and the encoding condition also had no significant interaction effect [*F*(2,70) = 0.16, *p* = 0.85]. Under the three kinds of encoding conditions, the recognition rates of positive trait adjectives were significantly higher than those of negative trait adjectives, the past collective self [*F*(1,35) = 10.31, *p* < 0.01, $\eta_{p}^{2}$ =0.23], the present collective self [*F*(1,35) = 11.61, *p* < 0.01, $\eta_{p}^{2}$ =0.25], and the future collective self [*F*(1,35) = 13.32, *p* < 0.001, $\eta_{p}^{2}$ =0.28].

### Discussion

Experiment 1 adopted the first-person perspective to probe into the positivity bias of the collective self in the past, present, and future. The results found that, in terms of explicit closeness, people had the same closeness both in the past-present collective self and the present-future collective self. There was also no difference in the frequency of people recalled the past and imagined the future at the level of the collective self. This study also found that the rating scores of positive trait adjectives of three types of the temporal collective self were significantly higher than those of negative trait adjectives, the response times of positive trait adjectives were significantly shorter than those of negative trait adjectives, and the recognition rates of positive trait adjectives were significantly higher than those of negative trait adjectives. These three indicators showed that people showed a positivity bias toward the temporal collective self from the first-person perspective, which verified Hypothesis 1. Experiment 1 also found that in terms of positive trait adjectives rating scores, the rating scores of the future collective self were significantly higher than those of the past collective self and the present collective self, while in terms of the difference in response times and recognition rates, people did not show a more positivity bias toward the future collective self. This result indicated that Hypothesis 2 was verified in trait adjectives rating scores, but it was not verified in terms of response times and recognition rates, which may indicate that Temporal Self-appraisal Theory cannot be directly extended to the field of the collective self after combining the results of the three indicators.

## Experiment 2

The findings in Experiment 1 showed the past collective self, the present collective self and the future collective self all showed a positivity bias from the first-person perspective. Experiment 2 used the third-person perspective to verify the results of Experiment 1, and preliminarily discussed the difference in the positivity bias of the temporal collective self between the first-person perspective and the third-person perspective.

### Methods

#### Participants

We randomly selected 37 undergraduate students from Guizhou Minzu University to participate in Experiment 2 by convenient sampling (including16 males and 21 females, and their average ages are 19.32 years old; SD = 1.00), and all participants signed the written informed consent, which was approved by the Ethics Committee of Guizhou Minzu University.

#### Materials, measurements, and procedures

The experimental materials and procedures of Experiment 2 were the same to those of Experiment 1. The difference was that the participants were asked to use the third-person perspective for the temporal collective self-reference processing. Reference to previous experimental operations in the third-person perspective (Yue et al., 2021; Bao et al., 2022), the specific three types of encoding conditions in Experiment 2 were the past collective self, (e.g., do others think that Chinese people were like this 5 years ago?), the present collective self (e.g., do others think Chinese people are like this now?), and the future collective selves (e.g., do others think Chinese people will be like this 5 years later?).

### Results

#### IOS rating and frequency rating

The results of the paired sample *t*-test indicated no significant difference between the past-present collective self (*M* = 3.95, SD = 1.55) and the present-future collective self (*M* = 3.92, SD = 1.77), *t*(36) = 0.12, *p* > 0.05.

The results of the paired sample t-test indicated no significant difference between the frequency of imagining future collective self (*M* = 4.38, SD = 1.16) and of recalling past collective self (*M* = 4.11, SD = 1.56), *t*(36) = 1.24, *p* > 0.05.

#### Trait adjectives rating scores

The 2 (valence: positive/negative) × 3 (encoding condition: past/present/future collective self) repeated measures ANOVA was performed for trait adjectives rating scores, whose results found the valence had the significant main effect [*F*(1,36) = 487.30, *p* < 0.001, $\eta_{p}^{2}$ =0.93], and the rating score of positive trait adjectives (*M* = 3.50, SD = 0.05) was significantly higher than that of negative trait adjectives (*M* = 1.62, SD = 0.05) (*p* < 0.05). The encoding condition had no significant main effect [*F*(2,72) = 1.25, *p* = 0.29, $\eta_{p}^{2}$ =0.03]. The valence and the encoding condition had no significant interaction effect [*F*(2,72) = 1.64, *p* = 0.21, $\eta_{p}^{2}$ =0.04]. Under the three kinds of encoding conditions, the positive adjectives rating scores were significantly higher than those of negative adjectives, the past collective self [*F*(1,35) = 173.86, *p* < 0.001, $\eta_{p}^{2}$ =0.83], the present collective self [*F*(1,35) = 405.31, *p* < 0.001, $\eta_{p}^{2}$ =0.92], and the future collective self [*F*(1,35) = 450.59, *p* < 0.001, $\eta_{p}^{2}$ =0.93].

#### Response times

The 2 (valence: positive/negative) × 3 (encoding condition: past/present/future collective self) repeated measures ANOVA was performed for response times during the encoding phase, whose results found the valence had the significant main effect [*F*(1,36) = 10.62, *p* < 0.001, $\eta_{p}^{2}$ =0.23], and the negative response times (*M* = 1797.11, SD = 92.86) were significantly longer than the positive (*M* = 1667.54, SD = 81.61) (*p* < 0.05). The encoding condition had the significant main effect [*F*(2,72) = 5.79, *p* < 0.001, $\eta_{p}^{2}$ =0.14], and the response times of the past collective self (*M* = 1839.99, SD = 100.04) were significantly longer than those of the present collective self (*M* = 1692.09, SD = 74.29) (*p* < 0.05) and of the future collective self (*M* = 1664.89, SD = 96.35) (*p* < 0.05). The valence and the encoding condition had no significant interaction effect [*F*(2,72) = 1.07, *p* = 0.35, $\eta_{p}^{2}$ =0.03]. Under the three kinds of encoding conditions, the negative response times were significantly longer than the positive, the past collective self [*F*(1,35) = 3.74, *p* = 0.06, $\eta_{p}^{2}$ =0.094], the present collective self [*F*(1,35) = 4.40, *p* < 0.05, $\eta_{p}^{2}$ =0.11], and the future collective self [*F*(1,35) = 11.83, *p* < 0.001, $\eta_{p}^{2}$ =0.25].

#### Recognition rates

The 2 (valence: positive/negative) × 3 (encoding condition: past/present/future collective self) repeated measures ANOVA was performed on the recognition rates during the recognition phase. The findings showed the valence had a significant main effect [*F*(1,36) = 9.18, *p* < 0.01, $\eta_{p}^{2}$ =0.20], the recognition rates of positive trait adjectives (*M* = 0.37, SD = 0.01) were significantly higher than those of negative trait adjectives (*M* = 0.33, SD = 0.01) (*p* < 0.01). The encoding condition had a significant main effect [*F*(2,72) =4.40, *p* < 0.05, $\eta_{p}^{2}$ =0.11]. The valence and the encoding condition had no significant interaction effect [*F*(2,72) = 2.49, *p* = 0.09, $\eta_{p}^{2}$ =0.07]. Under the three kinds of encoding conditions, the recognition rates of positive trait adjectives were significantly higher than those of negative trait adjectives, the past collective self [*F*(1,35) = 0.30, *p* = 0.58, $\eta_{p}^{2}$ =0.01], the present collective self [*F*(1,35) = 8.73, *p* < 0.01, $\eta_{p}^{2}$ =0.20], and the future collective self [*F*(1,35) = 8.801, *p* < 0.01, $\eta_{p}^{2}$ =0.20].

### Comparison of the results of experiment 1 and experiment 2

In order to explore whether there are differences in the positivity bias of the temporal collective self from different perspectives, we compared the results of experiment 1 and Experiment 2. The 2 (valence: positive/negative) × 3 (encoding condition: past/present/future collective self) × 2 (perspective: first-person/third-person perspective) repeated measures ANOVA were performed for trait adjectives rating scores, response times and recognition rates, respectively. The results showed that there was no interaction among three dependent variable indicators (valence, encoding condition, and perspective). Trait adjectives rating scores [*F*(2,142) = 0.46, *p* = 0.63], response times [*F*(2,142) = 0.22, *p* = 0.80], and recognition rates [*F*(2,142) = 2.40, *p* = 0.09]. The results showed that there was no difference between the first-person perspective and the third-person perspective in the positivity bias of the temporal collective self.

### Discussion

Based on Experiment 1, Experiment 2 adopted the third-person perspective to explore the positivity bias of the temporal collective self. It is found that consistent with the results in Experiment 1, compared with negative trait adjectives, positive trait adjectives rating scores of the past, present, and future temporal collective self were higher under the third-person perspective, response times were shorter, and recognition rates were higher. It indicated that the temporal collective self also had a positivity bias under the third-person perspective, which verified Hypothesis 3. Experiment 2 did not find any difference in trait adjectives rating, response times and recognition rates of the three types of the temporal collective self, then Hypothesis 4 was not verified. In order to explore the difference in temporal collective self from different perspectives (first-person perspective and third-person perspective), we also compared the results of experiment 1 and experiment 2, and no difference was found. This suggested that the distancing function of the third-person perspective found at the level of the individual self was not manifested at the level of the collective self.

## General discussion

This study adopted the adapted temporal collective self-reference paradigm. Through two experiments, it is found that in terms of trait adjectives judgement, response times and recognition rates, Chinese people show positivity bias in the past collective self, the present collective self and the future collective self in the trait word judgment, reaction time and recognition rate. This positivity bias is reflected in both the first-person perspective and the third-person perspective, and there is no difference in the positivity bias of the temporal collective self under the two personal perspectives. In addition, people did not show much of a more positivity bias toward the future at the collective level which is different from the expectations of Temporal Self-appraisal Theory.

This study extended the self-positivity bias to the temporal collective self for the first time, and found that no matter in the first-person perspective or the third-person perspective, people showed a more positivity bias toward different temporal collective self. It is mainly manifested that at different time points, people tend to rate positive trait adjectives as relevant to the collective self, while negative trait adjectives as irrelevant. The response times of positive trait adjectives judgment were faster than those of negative trait adjectives judgment. And people’s recognition rates of positive trait adjectives were higher than those of negative trait adjectives. These results suggested that the positivity bias of Chinese people’s temporal collective self is reflected not only in emotions but also in cognitive structures. This study found that people not merely tend to view themselves positively (Watson et al., 2007; Zhou et al., 2013), but also view their own group identity at different points in time with a positive attitude, indicating that collective self-relevant information has higher emotional significance for individuals (Zheng et al., 2018).

According to Temporal Self-appraisal Theory, Chinese people’s self-positivity may vary from point to point in time. People will show a greater positivity bias toward the future than the past and the present (Wilson and Ross, 2001). The results of this study suggested that Temporal Self-appraisal Theory may not be extended directly to the field of the collective self. Although the participants scored slightly higher on the trait adjectives judgment of the future collective self in the first-person perspective, they did not show a more positivity bias toward the future in response times, recognition rates, and trait adjectives judgment in the third-person perspective. In addition, the additional measurement of the IOS scale also showed that there was no difference in the closeness between the past-present collective self and the present-future collective self, and there was no significant difference in the frequency of people imagine the future and recall the past at the collective level. Existing studies have found that at the individual level, people think about the future much more than the past (Anderson and McDaniel, 2019; Yue et al., 2021), and the frequency of people think about the future is about three and a half times that of recalling the past (Baumeister et al., 2020), and they generally view their future self with a more positive attitude (Hershfield, 2011; Szpunar et al., 2012). These findings may suggest that though people spend more time thinking about the future, these thoughts and positive views of the future may be more shown at the individual level than the collective. Since this study is only a preliminary exploration, it needs to be further researched to what extent temporal self-appraisal can be extended to the collective level.

The study on the individual self has found that because of the distancing function of the third-person perspective (Sutin and Robins, 2008), people may evaluate their own behaviors more objectively, have lower emotional experience, and reduce egocentric bias, when viewing themselves from the third-person perspective, which is different from the first-person perspective (Berntsen and Rubin, 2006; Zhou et al., 2013). The results of this study found that people view the collective self in the third-person perspective, which has no difference from the first-person perspective. Based on Construal Level Theory (CLT), people take the individual self here and now as the reference point, forming different psychological distances. The representation of the near distance is more specific and contextualized, while of the far distance is more abstract and generalized (Trope and Liberman, 2003; Trope and Liberman, 2010; Henderson et al., 2011). This indicated that the representation of the collective self is relatively generalized, compared to the embodiment of the individual self. Therefore, regardless of adopting the first-person perspective or the third-person perspective, individuals’ different temporal collective self-representations are more abstract and generalized, and they ignore the internal state of the self (Pronin, 2008), activating a more general self-concept. Therefore, under the two personal perspectives, there is no difference in the positivity bias of different temporal collective self.

This study discussed the positivity bias of the temporal collective self in the first-person perspective and the third-person perspective, expanded the research field of the temporal collective self, and promoted the understanding of the temporal collective self. However, this study was only a preliminary exploration of this issue, and further researches should be needed. First, on selecting participants of the collective self, previous studies have found that the temporal orientations of different social groups might have differences, which may be driven by the inherent traits of the groups (Peetz and Wohl, 2019). This study only selected national members as the collective self-identity, so whether this result can be popularized to other collective self-identity needs further researches. Second, on the selection of time points, this study selected 5 years ago or 5 years later. Longer time may mean a greater change for the collective self, therefore, subsequent studies can try to explore a longer time span to probe into the stability of the results of this study. Third, the research on time orientation can be extended to the relational self. This study found that the temporal collective self-processing and the temporal individual self-processing were not the same, so what characteristics will the temporal relational self show? It is also worthy of the researchers to answer this question.

In conclusion, this study extended the research on the temporal self to the collective self. The main findings were that different temporal collective self show a certain positivity bias both in the first-person and the third-person perspectives, this positive bias did not vary from different personal perspectives, and people did not show a more positivity bias toward the future at the collective level. This study contributes to better understand the traits of the temporal collective self and its cognitive processing characteristics.

## Data availability statement

The raw data supporting the conclusions of this article will be made available by the authors, without undue reservation.

## Ethics statement

The studies involving human participants were reviewed and approved by the Ethics Committee of the Guizhou Minzu University. The patients/participants provided their written informed consent to participate in this study.

## Author contributions

CY constructed this study, drafted the initial manuscript, and designed the experiments. YL and CN carried out all the experiments in the study. DZ and HW processed and analyzed the sequencing data. CY, YL, and DZ checked and revised the data and the manuscript. All authors contributed to the article and approved the submitted version.

## Funding

This study was supported by the China Western Project of the National Education Science Planning Project, “The psychological mechanism and educational pathway of national identity of adolescents in China’s minority areas in the new era” Grant No. XMA200282.

## Conflict of interest

The authors declare that the research was conducted in the absence of any commercial or financial relationships that could be construed as a potential conflict of interest.

## Publisher’s note

All claims expressed in this article are solely those of the authors and do not necessarily represent those of their affiliated organizations, or those of the publisher, the editors and the reviewers. Any product that may be evaluated in this article, or claim that may be made by its manufacturer, is not guaranteed or endorsed by the publisher.
